# Techno-economic Analysis and Optimization of Intensified, Large-Scale Hydrogen Production with Membrane Reactors

**DOI:** 10.1021/acs.iecr.3c02045

**Published:** 2023-10-31

**Authors:** Dean M. Sweeney, Victor Alves, Savannah Sakhai, San Dinh, Fernando V. Lima

**Affiliations:** Department of Chemical and Biomedical Engineering, West Virginia University, Morgantown, West Virginia 26505, United States

## Abstract

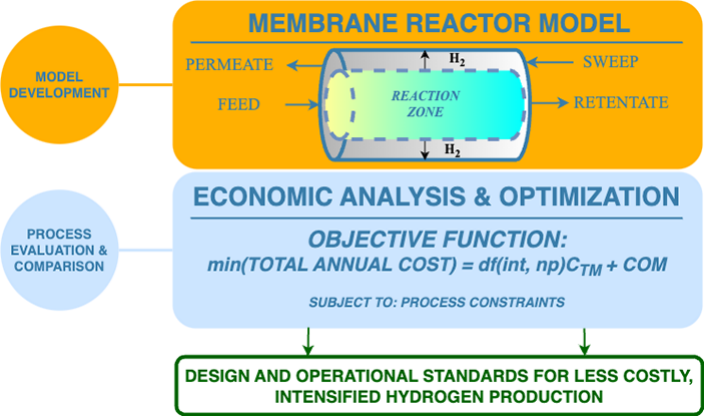

Steam methane reforming (SMR) currently supplies 76% of the world’s hydrogen (H_2_) demand, totaling ∼70 million tonnes per year. Developments in H_2_ production technologies are required to meet the rising demand for cleaner, less costly H_2_. Therefore, palladium membrane reactors (Pd-MR) have received significant attention for their ability to increase the efficiency of traditional SMR. This study performs novel economic analyses and constrained, nonlinear optimizations on an intensified SMR process with a Pd-MR. The optimization extends beyond the membrane’s operation to present process set points for both the conventional and intensified H_2_ processes. Despite increased compressor and membrane capital costs along with electric utility costs, the SMR-MR design offers reductions in the natural gas usage and annual costs. Economic comparisons between each plant show Pd membrane costs greater than $25 000/m^2^ are required to break even with the conventional design for membrane lifetimes of 1–3 years. Based on the optimized SMR-MR process, this study concludes with sensitivity analyses on the design, operational, and cost parameters for the intensified SMR-MR process. Overall, with further developments of Pd membranes for increased stability and lifetime, the proposed SMR-MR design is thus profitable and suitable for intensification of H_2_ production.

## 1 Introduction

Hydrogen (H_2_) is the main component in numerous industrial processes, such as ammonia and methanol synthesis, oil refining, and steel production.^1^ Due to its widespread usage, H_2_ production has tripled since 1975, reaching ∼70 million tonnes per year (MtH_2_/yr) in 2018.^1,2^ Its versatility in production and transportation makes it an attractive decarbonization technique for various industries, including power generation and fuel supply for vehicles and ships.^1^ The increased demand for H_2_ requires advanced developments for the scaleup of existing production technologies. Currently, 76% of H_2_ is sourced from natural gas, predominantly steam methane reforming (SMR).^1^ SMR involves the reaction between purified natural gas and superheated steam in a high-temperature and high-pressure reformer furnace, producing mainly carbon monoxide (CO), water (H_2_O), and H_2_. Due to the high temperatures (800–900 °C) of the system, traditional SMR requires ample heat duties provided by the combustion of fossil fuels. Consequently, global H_2_ production leads to CO_2_ emissions of ∼850 MtCO_2_/yr as of 2017. SMR’s large energy demand and carbon footprint introduce significant challenges when scaling-up its production to meet the increasing H_2_ demands while prioritizing decarbonization.^2^ Alternative low-carbon technologies, such as electrolysis, can mitigate these emissions, but currently are not economically competitive with traditional SMR. To address the challenges associated with the simultaneous scaleup and decarbonization of H_2_ technologies, the International Energy Agency (IEA) issued seven key recommendations, addressing H_2_’s role in long-term energy projects, its commercial demand, and the various production and transportation techniques.^1^ An essential recommendation outlined the development of current production facilities for less costly and less carbon-intensive H_2_ production. An alternative IEA study outlines the latter statement by simulating and costing the decarbonization of SMR plants with various carbon capture and storage techniques.^2^ The study showed significant capital and operating expenses tied to the integration of various carbon capture technologies. Therefore, addressing the second recommendation for less costly and efficient H_2_ production is essential to the simultaneous scaleup and decarbonization of SMR.

One particular development in SMR involves process intensification through H_2_ selective membrane reactors.^3^ The continuous equilibrium shift, caused by the removal of H_2_, significantly increases the efficiency of the traditional reformer and shift reactors. The lower-temperature operation (450–650 °C) promotes a three-reaction system, shown in reactions 1–3, with methane steam reforming (MSR), water–gas shift (WGS), and the overall reaction (OVR).MSR:
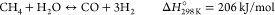
1WGS:
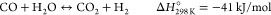
2OVR:
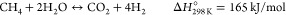
3

Multiple studies have shown the effectiveness of various membranes in separating H_2_ from a reaction zone and exploiting Le Chatelier’s principle, including its application toward water–gas shift reactions for coal gasification.^4−7^ Such membranes include dense metallic membranes, specifically palladium (Pd), and microporous ceramic membranes, including zeolites, silicas, and metal organic frameworks (MOFs).^8,9^ Ceramic membranes are limited by both their H_2_ selectivity and thermal stability, with rapid degradation in the presence of steam, a major component in SMR as a reactant and potential sweep gas.

Given the aforementioned limitations of ceramic materials, Pd-based membranes are currently most suitable for SMR operation with near-infinite H_2_ selectivity.^8,9^ However, pure Pd-membranes experience serious degradation at temperatures exceeding 500 °C. Alloying these membranes with other metals, such as copper (Cu), gold (Au), and platinum (Pt) increase their thermal stability up to 650 °C and prevent H_2_ embrittlement and membrane poisoning.^10−15^ In addition, intermediate layers for Pd membranes on porous metal supports, particularly stainless steel, prevent intermetallic diffusion and increase its high-temperature stability above 600 °C.^16^ These dense, high-temperature Pd-alloy membranes are already in commercial development with extremely high H_2_ selectivies (>1000) and fluxes (60–300 × 10^–3^ mol/(m^2^ s) at 100 kPa).^17^ The lifetime of the membranes is dependent on the operation, but it can be estimated to be ∼2–3 years.^18,19^ As for membrane costs, the support layers for the Pd-alloy membranes, which allow for thinner selective layers, contribute the majority of the cost, reported between $5000/m^2^ and $15 000/m^2^.^8,20−22^ However, as the number of manufactured membrane units increases, the future cost is expected to approach $1000/m^2^ to $5000/m^2^.^17,23^

Both experimental and modeling analyses examined Pd-MRs under operating conditions ranging from 450 °C to 700 °C, with pressure gradients of 10–20 bar and varying membrane thicknesses. In addition, the effect of a sweep gas within the shell side, usually H_2_O or N_2_, has been analyzed extensively.^5,6,24^ Typical CH_4_ conversions ranged from 85% to almost 100% with the latter achieved at higher temperatures, larger pressure gradients, and with the use of a sweep gas.^5,24^ With traditional SMR requiring temperatures exceeding 850 °C for 85% conversion, membrane reactors offer numerous benefits in their lower-temperature operation.

Beyond experimental studies, there is limited literature outlining the techno-economic performance of the membrane reactors within industrial SMR processes. Reported studies performed economic estimations for only small-scale H_2_ production, using capacity scale-up factors for estimations of equipment purchase costs.^21,25,26^ This provides only a crude estimation for a plant’s expenses, given the complexity of the various units, including the membrane reactor.^27^ These studies reported levelized costs ranging from $1.0/kg to $3.0/kg, compared to their reported conventional SMR costs of $4.0/kg to $4.5/kg. However, this is a slight inflation of SMR costs, which resides within the range of $0.5/kg to $2.0/kg, depending on the regional natural gas prices.^28^ Additionally, the relatively small production scale of these designs makes it difficult to conclude the feasibility of the SMR-MR process, when compared to conventional designs. One study presented a techno-economic evaluation of a 300 tons per day (TPD) SMR-MR plant.^29^ The study outlined the most effective cost factors in the SMR-MR process, including high operating temperatures and the membrane configuration, without performing topological or parametric optimizations on the SMR process. Additionally, the SMR-MR costs were not compared to conventional designs, and it was concluded that more-accurate economic evaluations need to be performed to validate and build on the results.

Altogether, there is a lack of significant developments with regard to techno-economic studies on large-scale SMR-MR processes. Furthermore, all previous optimizations utilized sensitivity studies to evaluate the operation and configuration of the membrane reactor. This limits the dimensionality of an optimization and prevents comprehensive insight into the performance of the membrane reactor within the entire process. To address this gap in the literature, this work integrates a Pd–Au membrane reactor into SMR H_2_ production to perform economic evaluations and comparisons to a conventional process. This includes detailed analyses of the fixed capital investment and manufacturing costs of both conventional and intensified H_2_ production processes. These cost figures are generated through comprehensive and validated costing methodologies, which minimize the assumptions and approximations employed in previous techno-economic studies and present a more-accurate costing model for both the conventional and intensified plants. The holistic costing model is utilized in the constrained, nonlinear economic optimization, which extends beyond the membrane’s operation and presents more realistic and optimal process set points for the entire conventional and intensified H_2_ processes. The rigorous costing and optimizations within this study are utilized to present design and operation standards for the application of membrane reactors to SMR. Altogether, this work provides a more accurate depiction of the competitiveness and inherent benefits of membrane reactors in the scaleup of lower cost H_2_ production.

The remaining sections of this article are organized as follows: An outline of the modeling approach for both H_2_ production processes, including an in-depth analysis of the membrane reactor, is provided in sections 2.1 and 2.2. This is followed by the capital and operating costing methodology along with the optimization approach outlined in sections 2.3 and 2.4. The performance of the optimized processes relative to their base counterparts is outlined for both the conventional designs and SMR-MR designs in Section 3.1. A final comparison of the optimized conventional and SMR-MR designs is utilized to conclude the superior process in Section 3.2. Section 3.3 outlines the design standards and considerations for the operation of the SMR-MR facility. A summary of the results and conclusions are outlined in section 4.

## 2 Modeling and Optimization Approach

### 2.1 Base Case Conventional Plant Model

**Figure 1:**
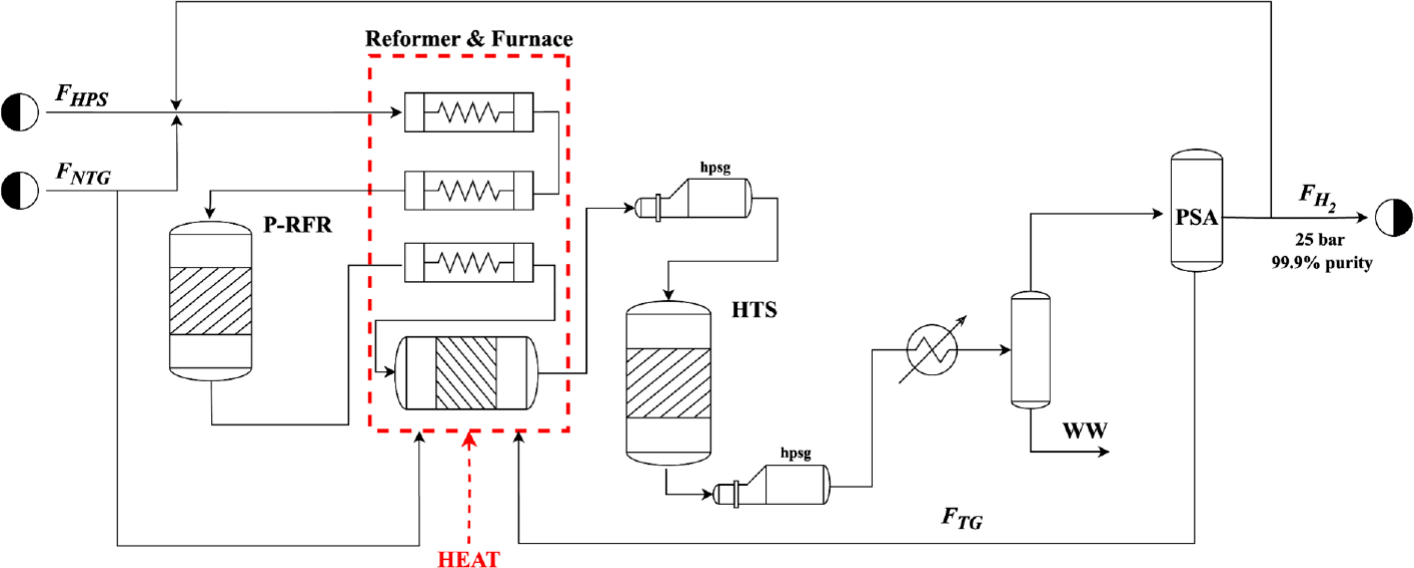
Conventional plant design of a standalone steam methane reforming merchant plant.

The conventional plant, displayed in Figure 1, is adapted from a standalone merchant plant, evaluated in an IEA report of H_2_ production technologies with carbon capture and storage.^2^ The process is built and simulated in Aspen Plus using set points and equipment specifications validated with literature.^30^ Pretreated natural gas (F_NTG_) and superheated steam (F_HPS_) at 400 °C and 42.0 bar are heated to 500 °C and fed at a steam:carbon (H_2_O/C) ratio of 2.8 into a prereformer (P-RFR), modeled as an adiabatic equilibrium reactor. The prereformer product is mixed with additional steam at a ratio of 2.8 H_2_O/C and then fed into the primary reformer, based on the Foster Wheeler Terrace Wall design.^2^ This unit is configured with a radiant section for reactions shown in reactions 1 and 2 and a convective section, used for preheating the prereformer and reformer feeds, as well as generating and superheating the steam. The reformer is modeled as a rate-based, multitube plug flow reactor (PFR). Reaction kinetics are based on Langmuir–Hinshelwood (L-H) kinetics for reactions 1 and 2.^31^ The reactions proceed at 925 °C in the reformer with a conversion of 85%. Downstream of the reformer is the high-temperature shift (HTS) reactor, modeled as a PFR at 400 °C also with L-H-based reaction kinetics for reaction 3.^31^

The conventional plant design includes significant heat integration for steam generation. Both the reformer furnace and HTS reactor are followed by waste heat boilers, used for high-pressure saturated steam generation (hpsg). In most cases, excess steam is produced and either sold as credit or is used for electricity generation. For the sake of simplicity, any excess steam is simply accredited in economic evaluations. The process syngas is cooled in a series of heat exchangers before the H_2_ is separated out in a pressure swing adsorption (PSA) unit, modeled as a component separator with 90% separation efficiency. The PSA tail gas (F_TG_) is mixed with supplementary natural gas for the reformer furnace. A small percentage of H_2_ exiting the PSA unit is recycled back to the feed. A rate of 100 000 N m^3^/h of H_2_ (F_H_2__) is produced at 37 °C, 25 bar, and 99.9% purity. Lastly, any wastewater (WW) is removed in the flash column preceding the PSA. Additional process specifications can be found in the referenced literature.^2,30^

### 2.2 Membrane Reactor and Base-Case-Intensified Plant Model

**Figure 2:**
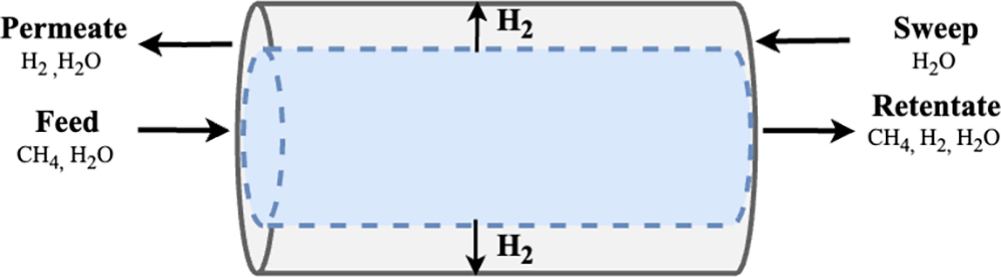
Membrane reactor schematic with counter-current steam sweep gas.

The MR system with the palladium membrane is modeled in Aspen Custom Modeler (ACM) as a tube and shell configuration, as shown in Figure 2. Model assumptions for the system include plug flow, steady-state, isothermal, and isobaric conditions, as typically assumed in MR literature.^4,32^ Additionally, H_2_ is assumed to be the only component to permeate the selective layer. Reactions 1–3 proceed within the inner tube over a packed bed of Ni/MgAl_2_O_4_ catalyst.^33^

The kinetic expressions for methane steam reforming are outlined in eqs 4–7.^7,26,31^
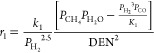
4
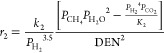
5
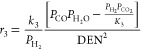
6
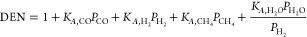
7

For each reaction, *i*, *r*_*i*_, *K*_*i*_, and *k*_*i*_ are the reaction rate, equilibrium constant, and rate constant, respectively. For each component *j*, *P*_*j*_ is the partial pressure and *K*_*A*,*j*_ is the temperature-dependent adsorption coefficient. Temperature-dependent reaction parameters, including *K*_*i*_, *k*_*i*_, and *K*_*A*,*j*_ are listed in Table 1 with the appropriate pre-exponential factors, activation energies, and enthalpies.

**Table 1:** Reaction Parameters Inputted into ACM for Methane Steam Reforming Kinetics^a^

**Kinetic Rate Constants** 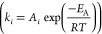			**Equilibrium Constants** 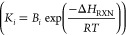		
*k_i_* (kmol/(MPa^0.5^ h kg_cat_))	*A*_*i*_	*E*_A_ (kJ/mol)	*K*_*i*_	*B*_*i*_	Δ*H*_RXN_ (kJ/mol)
*k*_MSR_	4.22 × 10^15^	240.10	*K*_MSR_	1.45 × 10^13^	220.20
*k*_WGS_	1.96 × 10^6^	67.13	*K*_WGS_	2.15 × 10^–2^	–37.72
*k*_OVR_	1.02 × 10^15^	243.90	*K*_OVR_	3.12 × 10^11^	182.4

**Adsorption Coefficients**		
component,*j*	κ_*j*_	Δ*H*° (kJ/mol)
CH_4_	6.64 × 10^–4^	–38.28
H_2_O	1.77 × 10^5^	88.68
H_2_	6.12 × 10^–9^	–82.90
CO	8.23 × 10^–5^	–70.65

a Data taken from ref (6).

Partial pressures for each component are calculated based on eqs 8 and 9, where *X*_*j*_ and *Y*_*j*_ represent a species mole fraction in the tube and permeate sides, respectively, and *P*_t_ and *P*_p_ respectively represent the total pressure in each section.
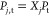
8
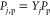
9

The flow rate (kmol/h) of species *j* is given by a differential mole balance on each species in the tube side, *F*_*j*,t_ and the permeate side, *F*_*j*,p_ in eqs 10 and 11. Also, ρ_b_ and ν_*j*_ are the catalyst bulk density (kg/m^3^) and reaction coefficient for component *j,* and respectively, *d* is the tube diameter.
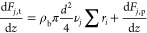
10
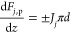
11

The flux (kmol/(h m^2^)) of H_2_ through the selective layer is given by Sievert’s law, shown in eq 12, where *Q*_*j*_ is the temperature-dependent permeability constant and δ is the membrane thickness. Because of the membrane’s infinite selectivity to H_2_, eq 12 is only applicable to H_2_ with all other component fluxes assumed to be zero.^8,9^
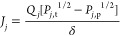
12

**Figure 3:**
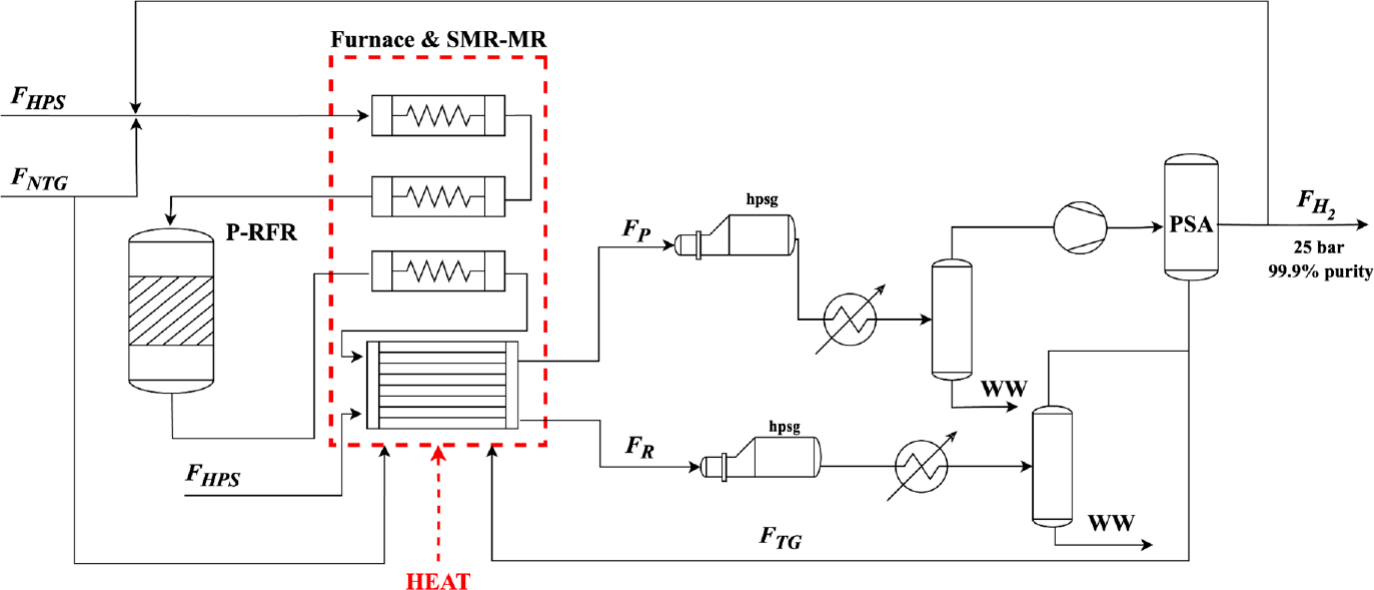
SMR-MR plant with a Pd–Au membrane integrated into the furnace heater.

The membrane reactor model is integrated into Aspen Plus as an ACM subroutine in replacement of the reformer and HTS reactors of conventional design. In the base case scenario, the reactor operates isothermally at 580 °C with pressures of 30 and 1 bar in the tube and shell sides, respectively. It has a respective total area of 450 m^2^ with a length of 6 m, diameter of 0.12 m, 200 tubes, and a membrane thickness of 10 μm. The full process flowsheet is shown in Figure 3.

A steam sweep gas (F_HPS_) is fed in both co-current and counter-current configurations at 2000 kmol/h. Due to the high separation efficiencies, the permeate stream (F_P_) contains most of the H_2_ mixed with the steam sweep gas. The steam from this stream is condensed out in a flash unit as wastewater (WW), and the vapor stream is fed into a compression cycle with interstage cooling. The H_2_ and residual water is fed into a PSA unit to achieve the necessary H_2_ purities. The final H_2_ product aligns with the conventional plant at 100 000 N m^3^/h of H_2_ at 99.9% purity, 37 °C, and 25 bar. The water in the retentate stream (F_R_) is flashed out and the residual CH_4_ and H_2_ in the tailgas (F_TG_) is fed to the furnace with supplemental natural gas. The intensified plant also includes waste heat boilers for steam generation on both the retentate and the permeate outlets.

### 2.3 Capital and Operating Costing Approach

The plant’s total annual costs (TAC), displayed in eq 13, was utilized to evaluate the economic performance of the conventional and intensified designs.^27^
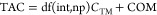
13

A plant’s TAC comprises its annuitized total module costs, *C*_TM_, and cost of manufacturing, COM. The *C*_TM_, shown in eq 14, is calculated as the cost of an expansion to an already existing facility with a capital discount factor, df(int,np). The plant lifetime (np) is 30 years, with an interest rate (int) of 10.0% and annual operation of 8000 h. The *C*_TM_ term is based on the bare module cost, *C*_BM_, which encompasses both direct and indirect expenses associated with the purchasing and installation of equipment and land. Project contingency and fees are defined as 15.0% and 3.0% of the total bare module cost, respectively. This is represented by a term α in eq 14 that takes the value of 1.18. A chemical engineering plant index factor (CEPCI) of 1.76 is employed for the year 2022.
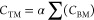
14
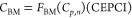
15
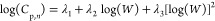
16

The bare module factor, *F*_BM_, accounts for all direct and indirect costs associated with equipment purchasing, including material costs for operating pressure and temperature, installation costs, and labor. The purchase cost, *C*_p,*n*_, is calculated based on the capacity factor for the equipment (*W*) and equipment parameters (λ_1–3_). For various equipment, *F*_BM_ and λ_1–3_ are extracted from the literature.^27^ The conventional plant capital accounts for the desulfurization unit, prereformer, reformer furnace, high-temperature shift reactor, PSA unit, waste heat boilers, and additional stainless steel shell and tube heat exchangers. Materials of construction (MOC) were selected according to the operating conditions of each piece of equipment. In particular, the high-temperature reformer furnace and boilers are made of specialty steel alloys with refractory lining.^34^ Stainless and carbon steel heat exchangers, compressors, and vessels are employed throughout the process. All equipment materials are costed with specialty material factors incorporated in the *F*_BM_ term.

The intensified hydrogen plant capital accounts for the same equipment, excluding the high-temperature shift reactor, a separate fired heater, and additional compressors. The membrane reactor is costed as a shell and tube heat exchanger with a derived costing function for its bare module cost, combining eq 15 and a specialized material factor for palladium.^27,35^ This is shown in eq 17, where *C*_p,Pd_ is the purchase cost of the membrane reactor, *A* the surface area, and Pd_cost_ is the membrane cost per unit area.
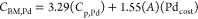
17

The COM term for the facility consists of the fixed manufacturing costs (FMC), direct manufacturing costs (DMC), and general manufacturing costs (GE).^27^ Estimations for values as percentages of specific costs are adapted from the literature.^27^ The FMC term, shown in eq 18, accounts for depreciation, local taxes and insurance, and plant overhead costs as 16.8% of the fixed capital investment (*C*_TM_) and 70.8% of the operating labor (*C*_OL_).
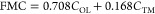
18

The DMC, shown in eq 19 is calculated as the summation of annual raw material (*C*_RM_), utility (*C*_UT_), waste treatment (*C*_WT_), and membrane replacement (*C*_MEM_) costs. The Pd–Au membrane replacement fee is evaluated as the material cost over its lifetime, as shown in eq 20. The current assumption for the membrane lifetime is 3 years, based on accepted standards and goals for Pd membranes.^11,18,19^ Fees, such as maintenance, repairs, and operating supplies, account for 6.9% of the *C*_TM_. Laboratory charges, direct supervisory and clerical labor, and patents and royalties are an additional 33.0% of the *C*_OL_ and 3.0% of the COM.^27^
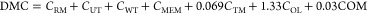
19
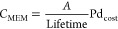
20

The general manufacturing costs (GE), shown in eq 21, consists of distribution and selling costs, along with research and development as 16.0% of the COM. Additionally, all administrative costs are accounted for as 17.7% and 0.9% of the *C*_TM_.^27^
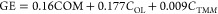
21

Finally, the COM of the plant is calculated as the summation of the FMC, DMC, and GE, shown in eq 22. Altogether, it accounts for all utilities, raw materials, treatment, and membrane replacement, in addition to indirect fees associated with the operation of all equipment. Additionally, the approximate cost savings from high-pressure steam generation are deducted from the overall costs, as *C*_hpsg_.
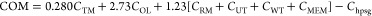
22

Adopted market values for various raw materials and utilities used in the process are displayed in Table 2.^27^ This includes natural gas, high-pressure steam, and approximate savings from steam generation. An average operator wage of $67 000/yr and 38 personnel is assumed for all plants, given the similarities between both the conventional and intensified processes.^2,27^ Total catalyst costs are ∼$340 000/yr for the feedstock pretreatment, prereformer, reformer, and shift reactor.^2^ The unit cost for the Pd–Au membrane is an average of the various values of metallic membranes reported in the literature between $5000/m^2^ and $15 000/m^2^.^20−22^

**Table 2:** Adopted Market Values for Process Utilities and Materials

item	unit price
Pd-Membrane, Pd_cost_	$10 000/m^2^
total catalyst	$340 000/yr
natural gas	$3.16/GJ
air	$0.5/100 S m^3^
high-pressure steam	$5.66/GJ
steam generated	$3.51/GJ
boiler feed water	$0.53/1000 kg
cooling water	$0.378/GJ
electricity	$0.0674/kWh
waste treatment	$41/1000 m^3^
operator wage	$67 000/yr

### 2.4 Optimization Approach

Economic optimization of both plants focused on the reactor configurations and plant set points for cost-effective, large-scale hydrogen production. The economic objective function, shown in eq 23, corresponds to the minimization of the plant’s total annual costs (TAC).
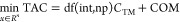
23subject to Aspen Plus simulation model equations
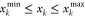

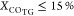

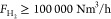

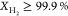


The large optimization problem contains 7910 variables for the conventional plant design and 11 552 variables for the MR plant design, as reported by the process simulator in the equation-oriented (EO) solution mode. The variable count, which consists of the process modeling variables as well as the decision variables, evidence the challenging nature of the optimization problem. In addition, decision variables, *x*, encompass plant set points for the conventional and intensified hydrogen production, including feed conditions and reaction operation, as shown in Table 3. The conventional plant has a total of seven decision variables with upper constraints on the reformer and shift reactor temperatures due to thermal stability of the vessel and catalyst. In addition, the H_2_O/C ratio entering both the prereformer and reformer is varied between 2.5 and 5, using natural gas and steam makeup streams. The lower constraint must be maintained to prevent catalyst deactivation. The physical configuration of the reformer and shift reactor units in the conventional plant were adapted from the literature and are not included in the optimization problem.^2^

**Table 3:** Decisions Variables and Their Bounds for Conventional and Intensified Plant Optimization Studies

decision variable, *x*	conventional	intensified
natural gas feed [kmol/h]	1000–5000	1000–5000
steam feed [kmol/h]	2000–10 000	2000–10 000
sweep flow rate [kmol/h]	–	500–10 000
prereformer H_2_O:C	2.5–5.0	2.5–5.0
reformer/MR H_2_O:C	2.5–5.0	2.5–5.0
prereformer temperature [°C]	500–950	500–650
reformer/MR temperature [°C]	800–950	500–650
shift temperature [°C]	300–430	–
MR tube length, *L* [m]	–	1–20
MR tube diameter, *d* [m]	–	0.01–0.5
MR tubes, *n*_*t*_	–	1–1000
**total**	**7**	**10**

The SMR-MR plant optimization problem contains 10 decision variables. The temperature of the membrane must be optimized for all three reactions with stricter bounds between 500 and 650 °C, due to the stability constraints of the membrane. However, the use of a sweep gas accounts for one degree of freedom (DOF), replacing the DOF loss from the removal of the shift reactor. In addition, the surface area of the multitube membrane reactor is incorporated into the optimization problem; this accounts for the tradeoff between MR performance and cost. Decision variables for this optimization include tube diameter (*d*), tube length (*L*), and number of tubes (*n*_t_). In addition, a constraint *L*/*d* ≥ 30 is imposed to maintain plug-flow operation within the reactor. Overall, both plants are constrained to a production rate of at least 100 000 N m^3^/h of H_2_ with 99.9% purity, *X*_H_2__, a CO content in the tailgas, *X*_CO_TG__, less than 15.0% for pollutant legislation, and furnace duties greater than or equal to the heating values of the tailgas to ensure efficient heat integration.^2^

Given the low density of H_2_, an additional constraint was placed on the H_2_ product pressure at 25 bar. The nonlinear, constrained optimization problem was solved using the sequential quadratic programming (SQP) solver built into the equation-oriented (EO) functionality within Aspen Plus.

## 3.0 Results

### 3.1 Conventional and SMR-MR Plant Optimizations

A total of five different SMR schemes were analyzed for their economic performance; these are summarized and detailed in Table 4. The base case conventional process reported a TAC of $101.0 million/yr with $100.0 million in capital expenses and $90.4 million/yr in manufacturing costs. The $100.0 million in *C*_TM_ is within 7.0% of the value ($107.6 million) reported from the IEA analysis.^2^

**Table 4:** Economic Performance of the Five Analyzed SMR Plants

iteration	*C*_TM_ [million $]	COM [million $/yr]	TAC [million $/yr]
**Conventional Process**			
base case	100.0	90.4	101.0
optimized case	97.4	87.6	97.9
**Intensified Process**			
base case co-current MR	82.7	100.7	109.5
base case counter-current MR	78.0	94.6	102.9
optimized counter-current MR	61.5	83.7	90.2

Additionally, the TAC is within 5.0% of the source used for validating the initial simulation, which compares the same conventional process to modularized SMR plants.^30^ The relatively small errors in *C*_TM_ and TAC are attributed to different assumptions for the cost estimations; nonetheless, they reinforce the validity of the methodology employed in this work.

Major expenses of the base case conventional plant are tied to the large heat duties and natural gas demand of the process. These include the natural gas and air utilities at $37.7 million/yr and $7.3 million/yr, respectively, along with $57.0 million in furnace capital. Although the steam demand is large within the plant, the heat integration present in the base case design produces a net steam credit of $550,000/yr. These expenses were targeted in the optimizations through minimizing the heat duty while increasing the CH_4_ conversion. Table 5 outlines the major utility and capital contributions to the base case plant, along with their approximate savings from economic optimizations. The steam cost encompasses steam feedstock and the approximate steam boiler savings.

**Table 5:** Major Contributions and Cost Savings after Optimizations for Conventional SMR Processes

process variable	base case cost	approximate savings
feedstock natural gas	$28.9 million/yr	$200 000/yr
supplemental natural gas	$8.8 million/yr	–
air	$7.3 million/yr	$300 000/yr
steam	–$550 000/yr	$420 000/yr
furnace	$57.0 million	$2.0 million

Within the conventional optimizations, the reformer and shift temperatures were increased to 950 and 430 °C, both of which are active constraints on their operation, due to material and catalyst stability. The total conversion within both the reformer and shift reactors was increased from 87% to 89% through the higher operating temperatures. In addition, the H_2_O/C ratio was lowered to 2.5 entering the prereformer and reformer. As a result, the feedstock natural gas and steam demands were lowered by $200 000/yr and $420 000/yr, respectively, which resulted in $1.0 million/yr in steam credit. Although the furnace temperature was increased to 950 °C, the heat duty of the furnace and boilers was lowered due to less steam and natural gas mass within the furnace. This resulted in a $300 000/year reduction in air utilities and a $2.0 million reduction in furnace capital costs. Despite the lower energy demand observed in the optimized case, the cost for supplemental natural gas remained approximately the same between both cases. This is due to the higher process conversion and H_2_ yield within the optimized plant, resulting in less CH_4_ within the tailgas. Overall, a reduction of $3.1 million/year in TAC is observed with a final value of $97.9 million/yr. The optimal process set points for the conventional process when compared to the base case set points are outlined in Table 6. The process flow diagram remains consistent with that in Figure 1.

**Table 6:** Process Set Points for Conventional SMR Processes

process set point	base case value	optimized value
natural gas feed [kmol/h]	1350	1321
steam feed [kmol/h]	3893	3430
prereformer H_2_O:C	2.8	2.5
reformer H_2_O:C	2.8	2.5
prereformer temperature [°C]	500	500
reformer temperature [°C]	925	950
shift reactor temperature [°C]	400	430

Within the intensified plants, the counter-current membrane configuration significantly outperformed the co-current system in its base plant performance. A TAC value of $102.9 million/yr was calculated for the counter-current system. This is approximately $6.6 million/year lower than the co-current case due to higher conversions and intrinsically better performance when using a counter-current sweep gas. Overall, the counter-current configuration compared well with the base case conventional design. Major expenses in the base case counter-current SMR-MR process included $34.5 million/yr and $5.6 million/yr in natural gas and air utilities, respectively. In addition, significant downstream compression is required for the intensified plant, which adds electricity costs, totaling $10.3 million/yr. Major capital expenses consisted of the furnace and compressors at $25.2 million and $15.0 million, respectively. An additional $13.4 million was added in membrane capital, which covers the installation of the membrane and all accompanying equipment. The overall cost distribution of this process, along with the general lack of membrane reactor design and operation literature, makes this a significant optimization problem.

Table 7 outlines the major utility and capital contributions to the base case SMR-MR flowsheet, along with their approximate savings from the economic optimization performed. The steam cost encompasses the steam feedstock, membrane sweep gas, and approximate steam boiler savings. Major active constraints present in the final design are the SMR reaction temperature at 650 °C and the H_2_O/C ratio at 2.5 entering both the prereformer and membrane reactor. The higher temperatures benefit the kinetics of the reactions presented within the system, but any further increase is limited by the thermal stability of the membrane.

**Table 7:** Major Contributions and Cost Savings after Optimizations for the SMR-MR Process

process variable	base case cost	approximate savings
feedstock natural gas	$26.6 million/yr	$336 000/yr
supplemental natural gas	$7.9 million/yr	$764 000/yr
air	$5.6 million/yr	$600 000/yr
steam	$1.0 million/yr	–$120 000/yr^a^
electric utility	$10.3 million/yr	$2.5 million/yr
furnace	$25.2 million	$3.1 milion
Pd–Au membrane	$13.4 million	$8.2 million
compressors	$15.0 million	$2.6 million

a Added production costs to the optimized SMR-MR case.

The CH_4_ conversion was increased from 96% to 99% through a temperature increase to the 650 °C constraint, as well as increased MR performance. This results in a $336 000/yr reduction in feedstock natural gas. Similar to the conventional plant, lower heat duties are present in the furnace, despite the high operating temperatures, resulting in a $764 000/yr reduction in supplemental natural gas and $3.1 million reduction in furnace capital costs. This, again, is due to the overall lower mass of natural gas and steam, both feedstock and sweep gas, which was lowered to 1152 kmol/h. Smaller sweep gas flow rates in the permeate stream boiler produces less steam credit, resulting in a $120 000/yr increase in steam costs.

The greatest reductions in the TAC are correlated with the configuration of the membrane reactor. The total membrane area was reduced from 450 m^2^ to 188 m^2^. However, the volume-to-surface area ratio (*V*/SA) was increased from 0.03 to 0.125 m as the diameter increased to 0.5 m, which is an active constraint on the system to avoid unusually large tube sizes. The increased *V*/SA value shows that the system benefits from an additional reaction volume within a given surface area for H_2_ flux. To satisfy the plug-flow *L*/*d* restriction, the length of the membrane was extended to 20 m with an *L*/*d* value of 40. Membrane tubes were reduced to 6 to lower the surface area. Overall, this resulted in a reduction in membrane capital of $8.2 million with significantly lower raw material and sweep utility demands associated with the increased membrane efficiency. Lastly, the permeate pressure maintained by the sweep gas was increased to 2 bar. Although this is a relatively small change, the lower compression ratios decrease electricity demands by $2.5 million/yr. Overall, the lower operating costs and both direct and indirect reductions in capital result in a $12.7 million/yr decrease in TAC with a final value of $90.2 million/yr. The optimal process set points for the intensified SMR-MR process when compared to the base case counter-current SMR-MR set points are outlined in Table 8. The process flow diagram remains consistent with Figure 3.

**Table 8:** Process Set Points for the Intensified SMR-MR Processes

process set point	base case value	optimized value
natural gas feed [kmol/h]	1250	1233
steam feed [kmol/h]	3605	3200
sweep flow rate [kmol/h]	2000	1152
prereformer H_2_O:C	2.8	2.5
MR H_2_O:C	2.8	2.5
prereformer temperature [°C]	500	500
MR temperature [°C]	580	650
MR surface area [m^2^]	450	188
MR *V*/SA [m]	0.03	0.125
MR *L*/*d*	50	40

### 3.2 Conventional vs SMR-MR Optimized Processes: Further Economic Comparisons

**Figure 4:**
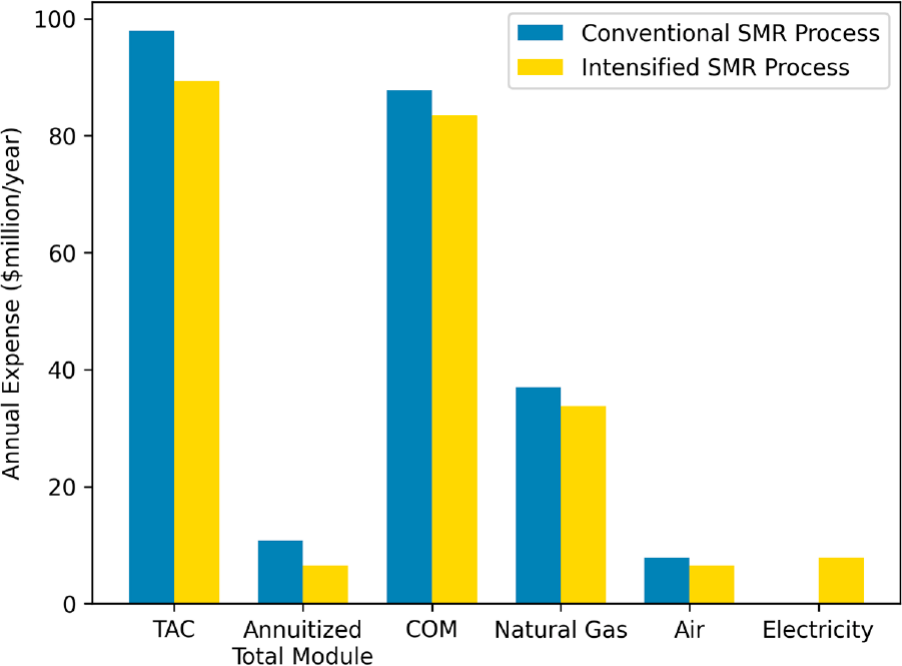
Annuitized economic analysis between the optimized conventional and intensified designs for a three-year membrane lifetime.

A TAC value of $90.2 million/yr ($1.25/kg) was generated for the intensified SMR-MR flowsheet. This is a 7.86% reduction in TAC when compared to the conventional optimized process TAC of $97.9 million/yr ($1.36/kg). Assuming a price of $4.0/kg of H_2_, a revenue of approximately $287 million/yr is obtained for both processes.^36^Figure 4 shows a breakdown of annuitized cost for both optimized plants. Despite the $18.0 million in compressor and membrane costs, significantly lower capital expenses were achieved with an intensified plant design. The furnace and waste heat boiler capital are decreased by $43.0 million due to lower-temperature operation. In addition, the removal of the shift reactor and lower demand on the PSA unit result in additional $6.0 million savings for the intensified design. When combined with the additional expenses associated with the capital, this leads to a total module reduction of $35.9 million or $3.8 million/yr.

In addition to the significantly lower capital, the combined fuel and feedstock natural gas usage is lowered by $3.2 million/yr, along with $1.4 million/yr in air costs due to increased conversions, higher H_2_ yields, and lower temperatures. These reductions are relatively insignificant when compared to the $7.8 million/yr in electricity associated with the intensified plant. However, the overall reduction in capital results in an additional $10.1 million/yr in manufacturing expenses associated with indirect fees, such as maintenance and repairs, plant overhead costs, depreciation, and taxes. Overall, the large reduction in capital with lower natural gas demand results in the $7.7 million/yr reduction in the optimized intensified plant TAC.

As proven, the SMR-MR process shows significant reductions in TAC when compared with the conventional process. However, there are assumptions tied to the membrane’s operation, including the approximate $10 000/m^2^ cost, three-year lifetime, and overall costing methodology.^20−22^ A break-even analysis between the optimized conventional and intensified designs was completed to account for these uncertainties. Approximate membrane costs per area calculated for lifetimes of 1–3 years to break even are displayed in Table 9.

**Table 9:** Break-Even Analysis for Various Membrane Lifetimes and Unit Costs

membrane lifetime	membrane cost [$/m^2^]
1 yr	25 000
2 yr	32 000
3 yr	35 000

As shown, break-even costs were calculated as $25 000/m^2^ to $35 000/m^2^, which are greater than the reported Pd-alloy MR costs between $5000/m^2^ and $15 000/m^2^.^20−22^ The high break-even costs signify the competitiveness of the SMR-MR process, even when accounting for price uncertainties and relatively short membrane lifetimes. The lower capital expenses and natural gas demand, along with its inherently safer design, make the SMR-MR process an attractive and economically viable H_2_ production technology for the future.

### 3.3 Sensitivity Analyses: Design, Operational, and Cost Parameters

As discussed above, SMR intensified with MRs shows significant advantages over conventional H_2_ production processes including superior economic performance. However, considerable analysis should be given to the design and operations of such a facility before implementation. The optimized SMR-MR process, with conditions outlined in Table 8, is further analyzed to present design and operating conditions that are essential to profitability over the optimized conventional SMR process.

**Figure 5:**
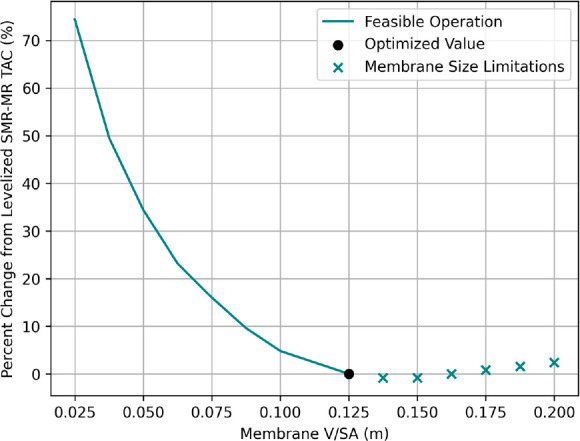
Percent change in SMR-MR TAC versus *V*/SA.

First, the MR is analyzed for design conditions that ensure elevated performance over conventional multitube plug-flow reactors. Proper design of the MR for intensifying an SMR process encompasses several parameters, including the volume-to-surface area ratio (*V*/SA), which is commonly used to understand reactor dynamics.^6,37^ The *V*/SA is manipulated through the MR diameter due to its squared effect on the reaction volume and proportional effect on the membrane surface area. Optimization of the MR design, as shown previously, favors larger *V*/SA ratios until membrane capital expenses outweigh the benefits of increased reaction conversions and membrane separation. Large-scale MR systems with relatively low catalyst costs, such as this, can require sizable tube diameters and *V*/SA ratios for increased reaction conversions and material processing.^38^ However, this relationship depends on the specific flux and reaction dynamics. Figure 5 details the relationship between the MR *V*/SA ratio considered and the changes in the optimized SMR-MR TAC value. It is important to note that when maintaining constant production rates under varying set points, the problem possesses a highly constrained and nonlinear behavior, which causes convergence limitations in the sequential modular solution mode. Hence, the system is analyzed under the constant feed and operating conditions outlined in Table 8 and a normalized TAC value, in terms of the production rate. This methodology remains consistent among all sensitivity studies to ensure that the results are analogous to the previous optimizations performed.

As shown in Figure 5, a *V*/SA ratio of 0.025 m causes over a 70% increase in the optimized SMR-MR TAC value. There are significant reductions in TAC when increasing ratios from 0.025 m to 0.125 m, attributed to higher CH_4_ conversions and membrane separation. However, just after the system’s optimized value of 0.125 m, the membrane capital expenses outweigh the increased MR performance, shown by the curve’s inflection at 0.150 m. Furthermore, there are size limitations placed on the membrane within this range to avoid unusually large tube sizes, given the *L*/*d* ≥ 30 restriction. This plug-flow restriction, satisfied by large tube lengths for sizable *V*/SA ratios, potentially causes capital expenses to outweigh MR performance prematurely. Therefore, even larger *V*/SA ratios with smaller lengths could provide additional cost reductions. Nonetheless, within the SMR-MR process, large *V*/SA designs maximize MR benefits over conventional processes with the optimal *V*/SA of 0.125 m yielding 10% higher CH_4_ conversions at lower operating temperatures, according to Tables 6 and 8.

**Figure 6:**
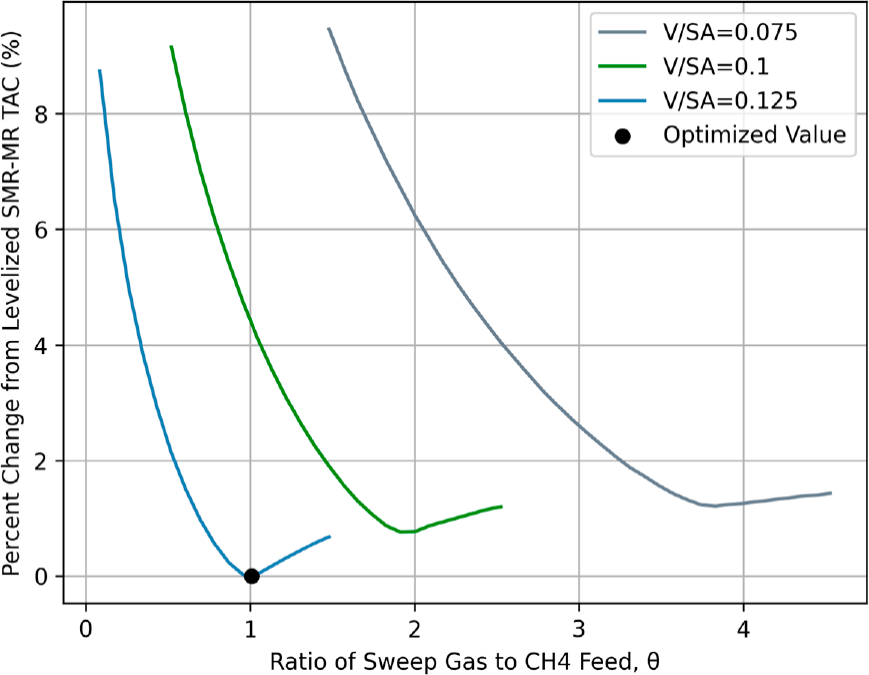
Percent change in SMR-MR TAC vs Θ at various *V*/SA ratios.

Following a design analysis of the MR, operating conditions for the SMR-MR process are considered for maintaining a lower COM than conventional technologies. Specifically, the optimized SMR-MR process has a 27% greater steam demand than the optimized conventional design, according to Tables 6 and 8. The increased steam demand results from the 1152 kmol/h of steam that is required as an MR sweep gas. However, the sweep gas enhances the MR separation capabilities, resulting in higher reaction conversions and H_2_ yields. Optimal usage needs to balance its impact on membrane performance and steam costs, as well as its effect on permeate waste heat boiler duties, which are essential to process steam production. This complex relationship is detailed in Figure 6, which examines the effect of sweep gas flow rates, relative to the MR CH_4_ feed (Θ), on the process TAC under various MR designs.

Throughout each design in Figure 6, sweep feeds increase for additional H_2_ separation until steam costs outweigh MR performance, resulting in three different optimal Θ values. A *V*/SA ratio of 0.125 m requires a Θ value of 1.0, yielding a permeate waste heat boiler duty of 60 GJ/h. Among *V*/SA ratios of 0.1 and 0.075 m, optimal Θ values increase to 1.9 and 3.8 to recover separation efficiencies. Additionally, the 20% and 40% increases in boiler duties at these optimal ratios offset the additional sweep utility costs, resulting in only small changes in TAC. However, despite the additional steam production, increasing Θ for less than optimal *V*/SA ratios has a degrading effect on the process TAC value, as shown by the curves in Figure 6 becoming more elongated at smaller *V*/SA ratios. This result emphasizes that both proper MR design and usage of a sweep gas are necessary to minimize its steam utility and maintain profitability over conventional designs.

**Figure 7:**
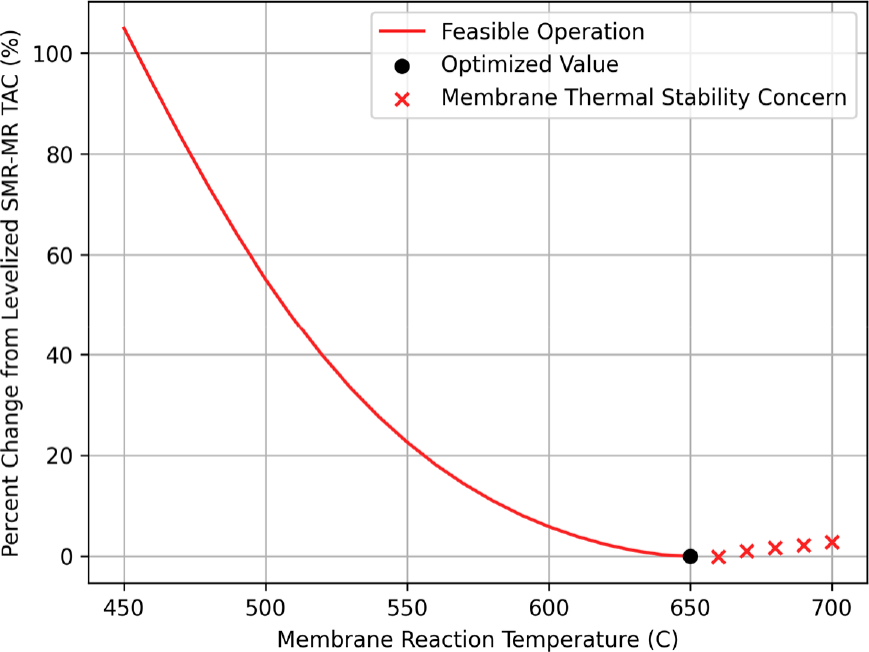
Percent change in SMR-MR TAC vs SMR reaction temperature.

The temperature of the SMR reaction was analyzed for its effect on the TAC. Due to the endothermic nature of SMR reactions, traditional operation, as shown previously, maximizes temperatures to over 900 °C for reasonable CH_4_ conversions. However, within the SMR-MR process, a temperature constraint of 650 °C is placed on its operation due to thermal stability issues of the Pd membrane. Therefore, optimal temperature set points will balance SMR reaction rates with furnace heat duty costs and with the thermal stability of the system. This relationship is outlined in Figure 7, which extends the range of operation beyond the 650 °C boundary to examine whether the thermal stability of the membrane poses significant limitations on the system’s performance.

As previously outlined, the SMR-MR process was optimized for a temperature of 650 °C, shown by the black dot in Figure 7. Although increased temperatures inherently benefit the system, there is only a slight decrease in TAC beyond 650 °C before the additional heat duties outweigh the increased reaction rates. Therefore, the SMR-MR process should be operated at its upper constraint to maximize profit while avoiding Pd thermal stability issues. Compared to the optimized conventional design, the SMR-MR process at the 650 °C set point has a 19% reduction in supplemental natural gas demands while maintaining a 6.1% reduction in feedstock natural gas usage, according to Tables 6 and 8. Although operating at temperatures less than 650 °C is somewhat desirable for reduced process heat duties, the low reaction conversions will cause exponential losses in profit. Overall, this indicates that the development of membrane materials for SMR applications should prioritize stability at ∼650 °C with a focus on maximizing membrane lifetimes under the SMR conditions.

**Figure 8:**
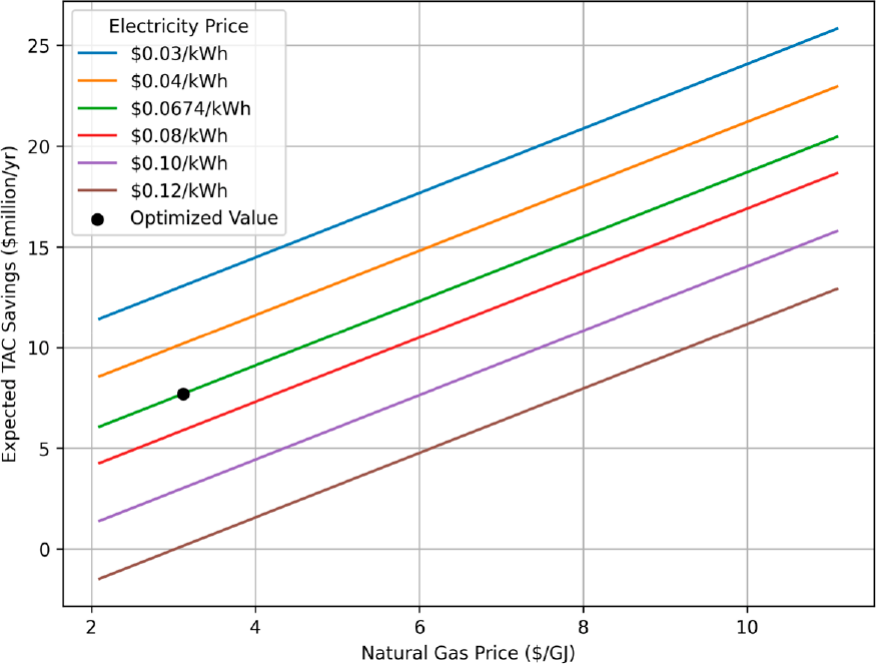
Expected TAC savings from the SMR-MR design over the conventional SMR process under variation in regional natural gas ($/GJ) and electricity ($/kWh) prices.

Following the MR optimizations for design and operating conditions, the proposed SMR-MR process is considered under variation in utility prices and their impact on the benefits of the design. Conventional H_2_ production suffers from large levelized TAC variations at $0.5/kg to $2.0/kg, due to its dependence on natural gas and the changes in gas prices by region.^28^ This effect is apparent in both the SMR-MR and conventional plant designs, as the feedstock and supplemental natural gas costs account for over 40% of the operating costs. Additionally, the SMR-MR plant requires 14.5 MW of electric utility, presenting a $7.8 million/yr difference in electric costs, relative to the conventional process. Therefore, exploring the effects of price variation is essential before continuing with either conventional or SMR-MR technologies. Figure 8 examines the expected TAC savings from implementing the SMR-MR design versus a conventional process at various natural gas and electricity prices.

As shown in Figure 8, high natural gas prices benefit the SMR-MR design. The opposite linear trend is observed with increased electricity prices. Additionally, the nominal price values of $3.16/GJ and $0.0674/kWh, used in the original optimization problem, reside at the lower end of the natural gas price spectrum, revealing that regions with higher natural gas prices could result in additional savings. Even with high natural gas and electricity prices exceeding $7/GJ and $0.08/kWh, respectively, which were observed within the United States in 2022, annual savings can exceed $10 million/yr.^39,40^ Nonetheless, Figure 8 not only outlines the economic spectrum of the SMR-MR process but also shows that careful consideration should be given to both the regional utility prices as well as their annual fluctuations before concluding the feasibility of the SMR-MR design to lower H_2_ production costs.

## 4 Conclusions

Five SMR plant designs were analyzed in this study, including two conventional designs adapted from the literature and three intensified membrane reactor designs. The conventional plant and counter-current membrane reactor design were optimized to obtain plant set points and a membrane configuration for large-scale H_2_ production. Overall, the conventional plant optimizations resulted in a savings of $3.2 million/yr with active constraints on the reformer and shift reactor temperatures. The intensified plant optimizations resulted in a savings of $12.7 million/yr. Active constraints were found in the steam feed, membrane configuration, and operating temperature, which are limited by the thermal stability of the membrane. Nonetheless, the optimization of the low-temperature SMR focused on maximizing conversion through higher temperatures for SMR kinetics and a membrane configuration with larger volume-to-surface area ratios.

The economic performances of the two optimized cases were compared to conclude the performance of the SMR-MR process. The intensified plant outperformed the conventional plant with a $7.7 million/yr reduction in TAC. To account for uncertainties with MR economics, a TAC breakeven analysis was performed between the optimized conventional and intensified designs. Break-even costs greater than $25 000/m^2^ were reported for short membrane lifetimes of 1–3 years when compared to the approximate membrane cost of $10 000/m^2^.

Following these studies, the optimized SMR-MR design was analyzed further to present design and operating conditions for MRs in SMR, including considerations for MR design, sweep gas utility, and operating temperatures of the system. This is coupled with a final consideration of the effects in the variation of natural gas and electricity prices on the benefits of the SMR-MR design over conventional technologies. Given the lower TAC values, inherently safer design, and less reliance on natural gas, the SMR-MR plant proves to be a competitive technology for the scaleup of low-cost, cleaner H_2_ production.
